# Role of Direct Oral Anticoagulation Agents as Thromboprophylaxis in Antiphospholipid Syndrome

**DOI:** 10.7759/cureus.19009

**Published:** 2021-10-24

**Authors:** Shreya Arora, Shaalina Nair, Rishab Prabhu, Chaithanya Avanthika, Sharan Jhaveri, Shilpa Samayam, Maanya R Katta, Pahel Agarwal

**Affiliations:** 1 Internal Medicine, Government Medical College and Hospital, Chandigarh, Chandigarh, IND; 2 Internal Medicine, California Institute of Behavioral Neurosciences and Psychology, Fairfield, USA; 3 Internal Medicine, Kasturba Medical College, Manipal, Manipal, IND; 4 Medicine and Surgery, Karnataka Institute of Medical Sciences, Hubli, IND; 5 Pediatrics, Karnataka Institute of Medical Sciences, Hubli, IND; 6 Internal Medicine, Smt. Nathiba Hargovandas Lakhmichand Municipal Medical College, Ahmedabad, IND; 7 Internal Medicine, Government Medical College Siddipet, Siddipet, IND; 8 Internal Medicine, Gandhi Medical College, Hyderabad, IND; 9 Internal Medicine, Bhaskar Medical College, Hyderabad, IND

**Keywords:** rheumatology & autoimmune diseases, thrombosis, hypercoagulable state, direct acting oral anticoagulant, antiphospholipid antibody (apla), thromboprophylaxis

## Abstract

Antiphospholipid syndrome (APS) is an autoimmune disorder that causes venous, arterial and small-vessel thrombosis, pregnancy loss, and premature birth. Cardiac valvular disease, renal thrombotic microangiopathy, thrombocytopenia, hemolytic anemia, and cognitive impairment are some of its other clinical symptoms. Antiphospholipid antibodies cause endothelial cells, monocytes, and platelets to become activated, as well as an increase in tissue factor and thromboxane A2. Complement activation might play a key function in pathogenesis.

Long-term oral anticoagulation is used to treat thrombosis, and individuals having arterial episodes should be treated quickly. Patients with systemic lupus erythematosus (SLE), as well as those with solely obstetric antiphospholipid syndrome, should get primary thromboprophylaxis. Obstetric care is based on a combination of medical and obstetric high-risk management, as well as aspirin and heparin therapy. Possible supplementary therapy for this condition is hydroxychloroquine. Statins, rituximab, and novel anticoagulant medicines are all potential future treatments for non-pregnant individuals with antiphospholipid syndrome. We aim to review the role of direct-acting oral anticoagulants (DOACs) as thromboprophylactic drugs in the treatment of APS in this article.

The treatment of venous thromboembolism has been transformed by a new class of DOACs. These drugs, such as rivaroxaban, function by inhibiting factor Xa directly. Not only do they have known anticoagulant actions, but they also obviate the need for dosage monitoring and modification, in contrast to warfarin.

We conducted an exhaustive literature search of PubMed/MEDLINE and Google Scholar Indexes using the keywords “Antiphospholipid syndrome,” “thromboprophylaxis,” and “oral anticoagulants” up to September 2021. We found that DOACs have been shown to be non-inferior to warfarin in a variety of anticoagulation situations in a number of high-powered clinical studies. In many hypercoagulable conditions such as APS, DOACs are quickly establishing themselves as first-line therapy. This article is focused on comprehensively reviewing the mechanism of action of DOACs, their role as thromboprophylactic drugs, risks and complications of DOACs, and comparing their efficacy with the standard treatment protocol and warfarin.

## Introduction and background

Antiphospholipid syndrome (APS) is a multisystem autoimmune disorder that is characterized by thrombosis (arterial, venous, or microvascular) and/or obstetric morbidity along with the presence of persistent antiphospholipid antibodies (aPL) in the serum. It is the commonest acquired hematologic cause of recurrent thromboembolic events [1]. It can occur as an isolated disease, called primary APS, or it can occur in association with other systemic autoimmune disorders (secondary APS). Younger adults of both genders, with a median age of 40 years, are seen to be affected predominantly by primary APS [2]. A marked female predominance has been observed in secondary APS, mainly when associated with systemic lupus erythematosus (SLE) [3]. “Catastrophic antiphospholipid syĺndrome” (CAPS) is another clinical entity defined by the occurrence of three or more new episodes of histologically confirmed organ thrombosis within a week in a patient with a history of APS [4].

The thrombotic events seen in APS are heterogeneous and can range from mild to potentially life-threatening recurrent episodes. The manifestations of arterial thrombi vary across a spectrum depending on the site of the thromboembolic event, the most common site being the cerebral vasculature, which usually presents in the form of a stroke or transient ischemic attack [5]. Occlusions in the retinal, coronary, renal, and mesenteric arteries can also occur. The venous thrombosis most commonly manifests as deep vein thrombosis of the lower extremities [6]. Other sites of venous thrombosis include the pelvic, renal, hepatic, superficial veins, portal, axillary and cerebral sinuses, and inferior vena cava.

The international classification criteria for antiphospholipid antibody syndrome states that APS can be defined by the presence of both clinical and laboratory criteria. The clinical criteria include arterial or venous thrombosis (which has to be confirmed by objective validated criteria using imaging studies or histopathology) and pregnancy morbidities, such as recurrent early miscarriages (before 10th week of gestation), late pregnancy losses, and/or premature birth due to severe preeclampsia or placental insufficiency. The laboratory criteria include the presence of these three antibodies: anticardiolipin (aCL), immunoglobulin G (IgG), and/or IgM antibodies by enzyme-linked immunosorbent assay (ELISA), anti-beta2 glycoprotein I antibodies (anti-β2GPI) IgG and/or IgM ELISA, and the lupus anticoagulant (LA) antibody. The presence must be confirmed on two or more occasions at least 12 weeks apart [7]. These antibodies are seen to have a direct pathogenic role as well, in addition to aiding in diagnosis [8]. The aPL profile, based on the type, titer, and the number of positive aPL tests defines the thrombotic risk of a patient. In addition to these antibodies, some of the other potential laboratory findings include thrombocytopenia, hemolytic anemia, prolonged activated partial thromboplastin time (aPTT), a history of false-positive venereal disease research laboratory test for syphilis, and low complement levels.

Anticoagulation has been the mainstay of treatment for thrombotic APS for years [9]. This involves heparin overlapping with warfarin or other vitamin K antagonists (VKAs). Therapy with standard-intensity warfarin is then continued indefinitely due to the high rate of recurrent thrombosis and the potentially devastating nature of these events, particularly arterial thrombosis. However, therapy with VKAs is often problematic, as they have a slow onset of action, require frequent monitoring with international normalized ratio (INR) with associated costs and burdens, and the monitoring itself is complicated as well, due to the variable responsiveness of reagents used in the INR test to lupus anticoagulants. VKAs have a very narrow therapeutic window and show numerous drug and dietary interactions. The dosing is affected by illness, changes in diet, and numerous interacting medications. Recurrences of thrombosis are reported in treated patients as well, despite anticoagulation with VKAs [10].

Due to these limitations, other therapeutic alternatives have been sought. There has been a recent interest in the use of direct oral anticoagulants for APS due to their convenience, lack of need for laboratory monitoring, fixed-dose administration, and decreased risk of bleeding [11]. They have a rapid onset of action, thereby diminishing the need to bridge anticoagulation with low molecular weight heparin (LMWH). Based on these features and a positive experience with these agents in other prothrombotic conditions, they have been proposed for secondary thromboprophylaxis (after a thrombotic event has occurred) in patients with APS. However, due to limited and contrasting data regarding their efficacy and safety, the use of direct-acting oral anticoagulants (DOACs) is still a matter of debate.

## Review

Epidemiology

Prevalence in Primary APS

Various studies have found the prevalence of aPL in the general population, with apparently healthy people, to be 1% to 5%. Furthermore, it increases with the aging process [12]. Another study stated that aPL is found to be in higher frequency in the young population [13]. The male-to-female probability may differ according to the type of APS, and it is known to be 1:3.5 for primary APS [5].

Prevalence in Secondary APS

According to research, aPL can be detected in up to 50% of SLE patients. Nonetheless, only one-third of these aPL-positive patients will eventually develop thrombotic events [14]. In a different study, the male-to-female probability for secondary disease associated with SLE is found to be 1:7 [5].

Epidemiology of APS

APS occurs only in a minor fraction of aPL-positive people. As per one study, five new cases per 100,000 persons per year was the approximate incidence of APS. The same study also found that 20 to 50 cases per 100,000 persons were the estimated prevalence [15,16]. When studied across the age groups, it was found that 85% of patients with APS are between 15 and 50 years of age, and according to sex, it is more widespread in women than men [5]. CAPS prevalence is less than 1% of all cases of APS [17-19]. An international registry known as CAPS Registry was developed in 2000 by the European Forum to systemize all the published case reports and the newly diagnosed cases from all over the globe [18].

Epidemiology of the Thrombotic Complications of APS

Multiple studies have indicated that nearly 30-40% of patients with aPL have a history of thrombosis and that venous incidents account for a majority of them while only 30% of the events are arterial [19,20]. Studies have also found that the most common arterial site affected is the cerebral vasculature [21]. In 29-55% of APS patients, venous thrombosis usually presents as DVT in the lower extremities within a follow-up period of six years [22-24]. An evaluated number of 11% of patients with myocardial infarction (MI), 9.5% of patients with DVT, and 13% of patients with stroke are documented with aPL positivity [17].

Epidemiology of Obstetric Complications of APS

Women with APS have a high risk of pregnancy loss from the 10th week of gestation [25]. In the general population, nearly 1% of the females attempting to have an offspring experience recurrent miscarriages, and 10% to 15 % of them are diagnosed with APS [26]. Complications of placental insufficiency, preeclampsia occur in about 50% of pregnant APS patients [27]; 25% of pregnant women with growth-restricted fetuses were found to have aPL in one study [28].

Pathogenesis

APS is an autoimmune disorder with characteristic features of recurrent thrombosis and pregnancy complications [29]. The aPL group has autoantibodies that interact with phospholipid-binding plasma proteins; the most significant one is β2-glycoprotein I (β2GPI) [30]. The plasma proteins (β2GPI) bind to phosphatidylserine, a phospholipid located on the inner surface of the cell membrane. This results in shifting phosphatidylserine onto the outer surface, inducing cell activation, clearance of apoptotic cells, and/or coagulation [31].

Pathogenesis of Thrombosis

One of the hallmarks of APS is thrombosis, and the components involved in inducing it are endothelial cells, platelets, monocytes, and the complement system.

Endothelial cells are activated as a result of anti-β2GPI antibody activity that causes the expression of adhesion molecules including vascular cell adhesion molecule-1 and E-selectin on their cell surfaces, resulting in a procoagulant condition. These antibodies also activate monocytes, which up-regulate tissue factor synthesis, activating the extrinsic coagulation pathway in the process. Platelets are also activated by anti-β2-GPI, resulting in an increase in glycoprotein IIb-IIIIa expression, thromboxane A2 production, and platelet factor-4 secretion [32]. These then induce a procoagulant condition [33]. aPL antibodies activate the complement cascade that stimulates the production of C3a, C5a, and the membrane attack complex (MAC) [32].

**Figure 1:**
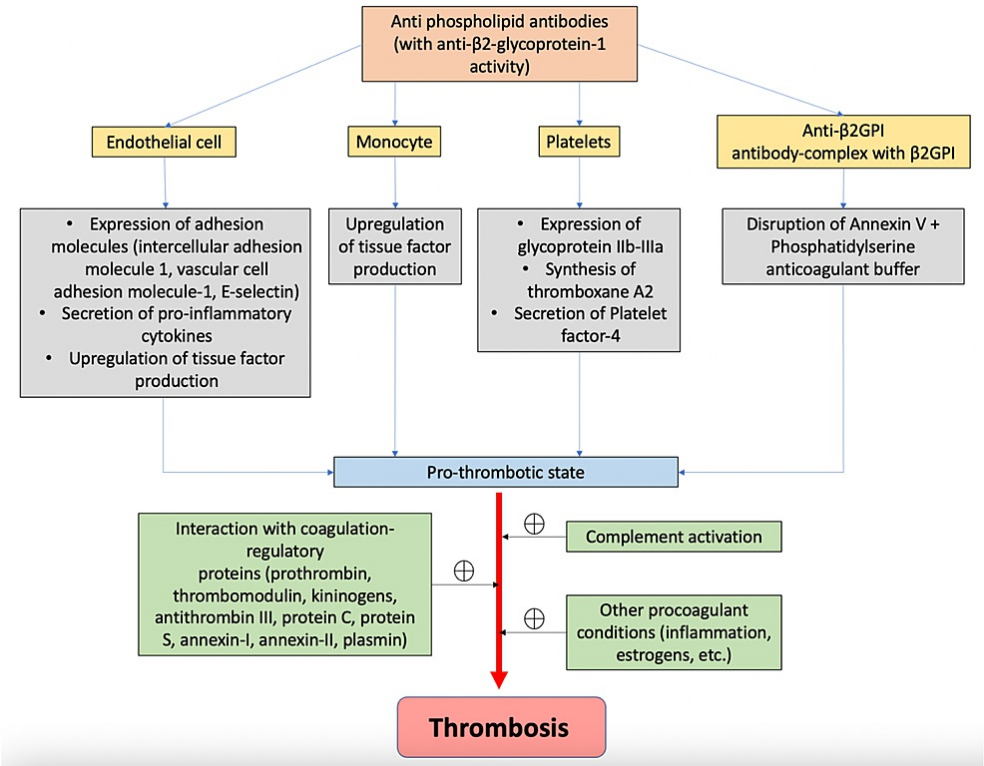
Pathogenesis of antiphospholipid antibody syndrome Anti-β2GPI: anti-β2-glycoprotein-1

In healthy individuals, Annexin A5 binds to phosphatidylserine, forming a buffer that limits the formation of procoagulant complexes. In APS patients, anti-β2GPI autoantibodies in complex with β2GPI disrupt this anticoagulant buffer, uncovering the procoagulant phosphatidylserine, therefore leading to thrombosis [34] (Figure 1).

It is observed that only a small percentage of patients who have aPL antibodies suffer from thrombotic complications, whereas the majority remain asymptomatic. This is explained by the "two-hit hypothesis," which suggests that the first hit (existence of aPL antibodies) is insufficient to cause clinical disease and that thrombosis requires a second hit, which might be estrogens, surgical procedures, trauma, or infections [32].

*Pathogenesis of Obstetric Complications*

In aPL positive pregnant women, many complications have been observed. The aPL antibodies could cause an intervention with annexin A5, which induces placental thrombosis and recurrent miscarriages [35].

β2GPI has an affinity to cytotrophoblast cells and can directly bind to them. Then, the anti-β2GPI antibodies bind and form antigen-antibody complexes. The formation of complexes can lead to the release of C5a [36], which consequently alters a variety of cell activities, that includes direct cellular injury, apoptosis or suppression of proliferation, and syncytia formation or defective invasiveness of the trophoblastic cells [32]. It has also been observed that aPL binds and reduces the secretion of human chorionic gonadotropin [37].

Mechanism of action of direct oral anticoagulants

DOACs work by inhibiting specific enzymes in the coagulation cascade. DOACs include direct factor Xa inhibitors (rivaroxaban, apixaban, and edoxaban), as well as dabigatran, the only drug that functions differently as a direct thrombin inhibitor (DTI), have all been approved by the Food And Drug Administration and are currently accessible for clinical usage [38]. It is also plausible that direct inhibitors of other clotting cascade enzymes may be developed in the coming years.

Factor Xa (FXa) is a clotting factor that plays a vital role in the coagulation pathway leading to thrombin production and clot formation. The direct FXa inhibitors work by blocking FXa, which is the initial step in the common pathway. Direct FXa inhibitors control thrombogenesis by selectively inhibiting FXa without requiring cofactors [39]. The inhibition occurs in a dose-dependent manner [40]. These drugs directly act on the FXa site on prothrombin (bound to Factor Va). These drugs also inhibit prothrombinase activity, hindering FXa from cleaving prothrombin to thrombin [41]. It has been illustrated that, when activated, one molecule of FXa can produce over 1000 molecules of thrombin [42]. They do not directly influence platelet aggregation induced by collagen, adenosine diphosphate, or thrombin, but by inhibiting FXa, they indirectly reduce clot formation induced by thrombin [43].

Thrombin (factor IIa) is the final enzyme of the clotting cascade that generates fibrin. It has a vital role in coagulation, activating other procoagulant factors, including V, VIII, XI, and XIII, and platelets [44]. Direct thrombin inhibitors (DTIs) bind to the active site of the thrombin enzyme (univalent DTIs) or two sites, the active site and exosite I, a positively charged portion of the thrombin molecule physically isolated from the active site (divalent DTIs) [44,45]. Both circulating and clot-bound versions of thrombin are functional. Because their area of binding to thrombin is not obscured by fibrin, DTIs can block both types of thrombin.

Dabigatran is a DTI and is a low molecular weight prodrug. Non-specific esterases in the liver and plasma convert it to its active form, dabigatran. Through ionic interactions, it binds directly to the active site of the thrombin. The inactivation of fibrin-bound thrombin and free thrombin is competitive and reversible. Dabigatran can stop thrombin-induced thrombus formation by preventing the conversion of fibrinogen to fibrin, positive feedback amplification of coagulation activation, cross-linking of fibrin monomers, platelet activation, and suppression of fibrinolysis in the coagulation cascade.

All of the direct FXa inhibitors are rapidly absorbed after oral administration. They have similar time courses for action. Penetration into the systemic circulation is initially rapid, with more than 90% of the drug reaching circulation 30 minutes after oral dosing. The impact of the intestinal biotransformation enzyme, P-glycoprotein (P-GP), on absorption is dose-dependent. Since the half-life of the active metabolite is longer than that of the parent compound, the duration of action is also dose-dependent. However, since multiple mechanisms eliminate all three drugs, there are inherent differences in their dosing regimens. The mean half-lives of dabigatran, rivaroxaban, edoxaban, and apixaban in hours(h) are 15.5 hours, 9 hours, 10 hours, and 12 hours, respectively. All four available DOACs are at least partially eliminated via the kidney, with Dabigatran having the most considerable extent of renal elimination (80%), less for edoxaban (50%), rivaroxaban (33%), and apixaban (22%), respectively [46]. These drugs are eliminated mainly by both hepatic metabolism and renal clearance. Apixaban is unique in that it has many elimination channels; it is eliminated by the hepatic (75% feces) and renal (25% urine) routes, through the bile, and directly through the intestines.

DOACs exhibit a linear pharmacodynamic profile with a known dose-response relationship. As a result, they can be given at a fixed dose and not be adjusted. The DOACs do not require routine laboratory monitoring for the same reason [41]. The clearance of DOACs from plasma is relatively quick once they are withdrawn, especially in patients with normal renal function.

Comparison with the efficacy of warfarin

The mainstay of treatment for APS is the use of long-term anticoagulants with VKAs. Warfarin is the standard treatment used for the management of thrombotic APS [47,48]. In patients with APS presenting with the first venous thromboembolism (VTE) event, the recommended anticoagulation is at INR of 2.5 (range 2.0-3.0) [49,50].

Although warfarin is the standard treatment for thrombotic APS, the management of APS is quite challenging. One of the several drawbacks includes the slow onset of action of warfarin [49]. It is also difficult to monitor patients due to several drug and alcohol interactions, narrow therapeutic windows, and constant monitoring of the INR to maintain the treatment level between 2.0 and 3.0 [51]. Warfarin reduces the Vitamin K-dependent coagulation factors (factors II, VII, IX, and X) and effectively affects all the phases of thrombin generation [51].

Warfarin also affects the clotting time, thus affecting the INR [52]. Due to the different responsiveness of the thromboplastin reagents to LA and the prolonged clotting time when treated with warfarin, the monitoring of INR for the APS patients further complicates. This may become an obstacle to diagnosing APS and monitoring the aPL status of the already present diagnosis when the patient is on warfarin [53].

The limitations and drawbacks of warfarin have driven the need for searching for new anticoagulant agents. The novel non-VKA anticoagulants or DOACs are considered as alternative options to VKA. DOACs may have a few advantages over the VKAs due to fixed doses, lower occurrence of bleeding incidents, rapid onset of action, lack of dietary restrictions, and interactions with drugs and alcohol, thereby improving the patient's quality of life [54,55].

It has been established that both warfarin and rivaroxaban are effective in patients with VTE who receive standard intensity treatment [56,57]. A lower rate of intracranial and fatal bleeding was also recorded in patients administered with rivaroxaban than those administered with warfarin [58]. DOAC administration is safer as the bleeding rate of 3.6% per year, whereas the bleeding rate for warfarin is up to 10% per year [59].

Results From RCTs Comparing the Efficacy of Rivaroxaban Versus Warfarin

The TRAPS (Trial of Rivaroxaban in Antiphospholipid Syndrome) study was conducted to compare the efficacy of rivaroxaban and warfarin. This trial was stopped early due to the increased thrombotic events that occurred in the rivaroxaban group. High thrombotic risk is seen in patients with triple-positive aPLs [60]. Four of 11 patients with recurrent thrombosis in the rivaroxaban group had a history of arterial thrombosis events.

In the study by Cohen et al. (rivaroxaban in antiphospholipid syndrome - RAPS trial), it is seen that there is a significant increase in endogenous thrombin potential (ETP) and time to peak thrombin generation in most patients following the change from warfarin to rivaroxaban [61]. The RAPS study included the patients with isolated venous thrombosis and showed no major bleeding or thrombosis event after 210 days of rivaroxaban and warfarin.

Two other RCTs showed an increase in thrombotic events when APS patients were changed from warfarin to rivaroxaban [60,62]. Results from a meta-analysis point out that there is a threefold chance of thrombosis in patients using DOACs who have a history of arterial thrombosis [63].

Based on the RCTs of the European League Against Rheumatism (EULAR), the British Society of Haematology (BSH) has stated indications and guidelines for the management of APS using DOACs [64]. Although guidelines suggest against the use of DOACs in patients with arterial thrombotic events and APS patients with triple positivity, DOACs may be considered for use in cases of venous APS patients and non-triple-positive APS patients [65]. In case of a recurrent thrombotic event, American Society of Hematology (ASH) guidelines have suggested the use of LMWH over the DOACs [66].

The increased thrombotic events in rivaroxaban may be due to the absence of a specified treatment regimen for rivaroxaban in APS patients. Rivaroxaban undergoes peak-to-trough fluctuations during the treatment while the treatment effect of warfarin is constant [67]. Evidence shows that BID dosing of Apixaban and Dabigatran is associated with lower trough concentrations [68]. These drugs give rise to a more stable anticoagulation level in the blood, thereby reducing the risk of thrombotic events [69].

Although there are some contraindications regarding the use of DOACs in triple-positive APS, it may be considered in patients intolerant to VKAs. The ongoing trials for DOACs: ASTRO-APS (Apixaban for Secondary Prevention of Thromboembolism Among Patients With AntiphosPholipid Syndrome) and RISAPS (RIvaroxaban for Stroke Patients With AntiPhospholipid Syndrome). In the ASTRO-APS study, patients with a history of arterial thrombosis were excluded from increased arterial thrombotic events due to DOACs treatment [63]. These studies may pave the way for the use of DOACs based on the clinical phenotype of APS.

An overview of trials comparing DOACs and VKAs across a range of parameters is shown in Table 1.

**Table 1:** Results from the clinical studies on the use of direct oral anticoagulants for antiphospholipid antibody syndrome APS: antiphospholipid syndrome, ASTRO-APS: apixaban for secondary prevention of thromboembolism among patients with antiphospholipid syndrome, BID: twice daily, ETP: endogenous thrombin potential, OD: once daily, RCT: randomized controlled trial, RAPS: rivaroxaban in antiphospholipid syndrome, TRAPS: trial of rivaroxaban in antiphospholipid syndrome, VKA: vitamin K antagonist, VTE: venous thromboembolism

Study and reference	Type of study	Sample size (DOAC/VKA)	DOAC	VKA	Treatment/follow-up time	Primary endpoint	Thrombosis (DOAC v/s VKA)	Study outcomes
RAPS trial (Cohen et al.) [61]	RCT	116 (57/59)	Rivaroxaban/20 mg OD	Warfarin/2.5	210 days	Changes in ETP	0% vs 0%	ETP is higher in the Rivaroxaban Group. Rivaroxaban does not reach the non-inferiority threshold in APS.
Goldhaber et al. [70]	RCT	151 (71/80)	Dabigatran/150 mg BID	Warfarin/2.5	Up to 36 months	VTE/VTE-related deaths	4.2% vs 5.0%	The efficacy of Dabigatran is not affected by APS or Thrombophilia.
Ordi-Ros et al. [62]	RCT	190 (95/95)	Rivaroxaban/20 mg OD Or 15 mg OD if creatinine clearance is 30-50 mL/min	Warfarin/2.5 (2.0-3.0)	36 months	Thrombosis	11.6% vs 6.3%	Recurrent thrombosis is 11% in rivaroxaban group and 6.3% in VKA group. Rivaroxaban does not show non-inferiority to VKAs in thrombotic APS.
TRAPS (Pengo et al.) [60]	RCT	120 (59/61)	Rivaroxaban/20 mg OD Or 15 mg OD if creatinine clearance is 30-50 mL/ min	Warfarin/2.5	611 days	Incidence of thromboembolic events, major bleeding, vascular deaths	12% vs 0%	The study was stopped due to increased events in the Rivaroxaban group.
ASTRO-APS (Woller et al.) [71]	RCT	30	Apixaban/2.5 mg BID (in 2015), 5 mg BID (since 2016)	Warfarin/2.5	Ongoing trial Follow up: 13 months	Arterial or venous thrombosis	NA	Active trial. The protocol was changed twice to 5 mg BID instead of 2.5 mg BID exclusion of patients with prior arterial thrombosis.

Current treatment protocol for antiphospholipid syndrome

Acute Thrombotic APS

Patients who develop the first episode of acute venous thrombosis are treated with unfractionated heparin or LMWH, which is then changed to a VKA, commonly warfarin, and is continued life-long. They should be frequently followed up to obtain a target INR of 2.0-3.0. Patients who have developed arterial thrombosis should ideally be given aspirin and VKA and followed up to achieve a target INR of 2.0-3.0. If a high-risk patient develops thrombosis, management should include anticoagulation with high-intensity VKA and a target INR of 3.0-4.0 [72]. In a subset of patients found to be triple-aPL positive, DOACs are not recommended for use as thromboprophylaxis in APS [73].

Recurrent Arterial and Venous Thrombosis in APS

In treating thrombosis refractory to initial anticoagulation, the first treatment measure is to ensure that the patient has achieved a target INR and a therapeutic factor X level [74]. To prevent the development of recurrent thrombosis, the patient's INR should be 2.5-3.0, and they must be supplemented with low-dose aspirin if the patient has cardiovascular risk factors [75]. In addition, for patients diagnosed with recurrent thromboses and who are currently on optimal warfarin therapy, a higher INR target of 3.0-4.0 should be targeted with or without low-dose aspirin, hydroxychloroquine, or low-dose aspirin, hydroxychloroquine a statin [76]. In patients who have INR >3 but still develop recurrent thrombosis, LMWH should be used instead of VKA [77].

Obstetric APS

The usual treatment would be a combination of heparin that can either be unfractionated heparin or LMWH given in prophylactic or intermediate doses with a combination of low dose aspirin, 75-100 mg daily [78]. Two more studies also support this and mention that the heparin administration should be continued 6-12 weeks in the postpartum period [79,80]. Warfarin has teratogenic effects, and women who were previously on VKA must be switched to LMWH ideally before or after the pregnancy is detected [72]. A combination of aspirin and therapeutic dose LMWH should be given to women diagnosed with APS and to those with prior episodes of arterial or venous thrombosis. In addition, a recent study by Empson et al. demonstrated evidence that aspirin alone does not improve pregnancy outcomes in APS [81].

Asymptomatic APS

Not many studies suggest the use of anticoagulation or antiplatelet therapy for primary thromboprophylaxis for asymptomatic aPL in patients regardless of SLE status [82]. Therefore, there is no role of aspirin as thromboprophylaxis in asymptomatic APS patients as the annual risk of developing acute thrombosis is low [83]. Aspirin is only recommended as primary thromboprophylaxis in patients with cardiovascular risk factors, as suggested by the 13th Congress on Antiphospholipid Antibodies [74]. Furthermore, patients with asymptomatic APS are started on LMWH or aspirin only in high-risk periods such as surgery and hospitalization [21]. Reversible risk factors such as obesity, smoking, and combined oral contraceptives should be minimized.

Catastrophic APS

CAPS is a rare variant of APS. CAPS involves the development of thrombosis in multiple organs and a storm of cytokines developing acutely, which is confirmed with evidence of multiple thromboses on a histopathological report and titer evidence of increased aPL antibodies. Treatment for CAPS includes therapeutic anticoagulation and additional therapies such as plasmapheresis, intravenous immunoglobulin (IVIG), and/or immunomodulatory agents to suppress the cytokine storm. If the patient develops life-threatening complications, treatment involves anticoagulation with intravenous heparin, high dose corticosteroids, and IVIG or plasmapheresis. However, if the patient is not in a life-threatening condition, treatment is with intravenous heparin and high-dose corticosteroids [84].

Other Therapeutic Modalities

Hydroxychloroquine (HCQ) is usually the prescribed treatment for patients with SLE. It is used primarily to reduce inflammation and modulate the immune system. It also reduces the risk of arterial and venous thrombosis in patients with SLE, with and without APS, as reported in a study by Petri [85]. In addition, patients with APS have aPL antibodies, which will activate platelets exposed to low-doses of adenosine diphosphate (ADP) or thrombin. Therefore, a study by Espinola et al. has concluded that HCQ will inhibit the expression of GPIIb/IIIa by platelets when exposed to a thrombin receptor agonist or aPL antibodies [86].

Statins have been shown to reduce levels of tissue factor (TF) induced by tumor necrosis factor (TNF-alpha) and lipopolysaccharide (LPS) on endothelial cells (ECs). A study by Meroni et al. concludes that rosuvastatin can reduce episodes of venous thrombosis in healthy individuals without high cholesterol. It also can inhibit anti-β2GPI antibodies and up-regulation of TF expression on ECs [87]. Another study by Ferrara et al. concluded that Fluvastatin inhibits up-regulation of TF expression by aPL antibodies on ECs [88].

Monoclonal antibodies are another therapeutic modality to be considered. Rituximab can be used to manage thrombocytopenia, hemolytic anemia, and skin ulcers associated with APS, but it does not alter the risk of developing thrombosis [89]. Another study by Kahn et al. concluded that inhibition of B cell-activating factor (BAFF) prevented the onset of APS and prolonged survival in autoimmune mice [90].

In conclusion, APS is an autoimmune disorder characterized by an increased tendency of developing arterial and venous thrombosis that can lead to multiple complications such as stroke, deep vein thrombosis, pulmonary embolism, and recurrent miscarriage. The goal of treatment is to prevent thrombosis, and we conclude that anticoagulation with LMWH bridged to VKA is the most crucial step in management. Management in specific high-risk groups can be supplemented with aspirin. HCQ, statins, and monoclonal antibodies are treatment modalities that can be explored in patients with refractory arterial and venous thrombosis.

Risks and complications of direct oral anticoagulants

Hemorrhagic Complications

An important complication with any anticoagulant is bleeding. The overall risk of bleeding complications with DOACs at the therapeutic dose is the same as that of VKAs, and the rates of intracranial hemorrhage are lower in the atrial fibrillation (AF) studies. The risk of gastrointestinal bleeding and potential drug interaction should be considered in a patient with APS who is on an antiplatelet and anticoagulation. Also, the risk should be paid attention to in people with SLE and autoimmune diseases where other medications could be considered including non-steroidal anti-inflammatory drugs (NSAIDs) and steroids [2]. Since APS is more common in women (the female to male ratio is around 5:1) [59], the incidence of vaginal bleeding should be considered. It is the most common complication seen in women on oral anticoagulation [91], witnessed especially more with FXa inhibitors than VKAs [92].

A meta-analysis study was conducted to compare the relative odds of fatal bleeding in patients on DOAC versus VKA therapy, where it reported 4056 major first-time bleeding events [93]. The calculated odds ratio for a fatal bleed in case of a significant bleeding event favored the DOACs, which was 0.65 (95% CI: 0.52, 0.81).

In a study conducted by Nagarakanti et al., the incidence of a major bleed with rivaroxaban and VKAs was similar (3.6% vs. 3.45%) [94]; and as per Romualdi et al., in the Einstein-extension study, the incidence was lesser (0.8% vs. 1.2%) [95]. But the risk of mucosal bleeding (GI bleed, genitourinary bleed, epistaxis) and anemia was higher in long-term use of rivaroxaban than VKAs [93]. No major bleeding event in women was observed except worsening menorrhagia in two women [96].

In the case of Dabigatran's safety profile, the RE-COVER and RE-COVER II trials compare its incidence of bleeding with that of VKAs, where it reported that incidence of any bleeding event is significantly lower with dabigatran [hazard ratio (HR) 0.70; 95% CI: 0.61, 0.79], as well as the incidence of major bleeding events and clinically relevant non-major bleeds (HR 0.62; 95% CI: 0.50, 0.76). Dabigatran was shown to be as effective as warfarin for the acute treatment of VTE [97].

The management of bleeding is the same for whether it is caused by DOACs or VKAs [98-100]. We have to note the time since the last dose of DOACs and the use of any other medications that can increase the risk of bleeding, for example, antiplatelet agents and NSAIDs, or any drugs which interfere with its metabolism. Hemodialysis plays an important role in reversing the anticoagulation effect of dabigatran because of its low protein binding capacity [101,102]. On the contrary, dialysis is not useful in reversing the effects of rivaroxaban and apixaban because of high protein binding capacity (both of them with 85%). Oral activated charcoal has been shown to reduce the absorption of dabigatran within three hours in an in-vitro study [98]. At present, the mainstay of treatment for bleeding due to DOACs is supportive measures, which include withdrawing the drug, mechanical compression, and surgical hemostasis and transfusion support. These agents disappear from circulation rapidly due to their short half-lives, unlike VKAs [98-100].

Currently, there are no specific antidotes in the market for the DOACs, so general hemostatic agents should be used to reverse the anticoagulation. These include prothrombin complex concentrate (PCC), activated prothrombin complex concentrate (aPCC), and recombinant factor VII activated (rFVIIa). Several studies in healthy individuals have shown that PCCs can reverse the effects of apixaban and rivaroxaban and not dabigatran [98-105]. aPCC seems promising for reversal of DOACs, especially in the case of dabigatran, but aPCC has an increased risk of thromboembolism. A monoclonal antibody fragment (idarucizumab, aDabiI-Fab) is tested to determine whether it can reverse the effect of dabigatran. An initial analysis from the RE-VERSE AD trial showed that idarucizumab completely reverses the anticoagulant effect of dabigatran [106]. Early results in healthy individuals show that adexanet was effective in reversing the effects of apixaban and rivaroxaban. Adexanet is a recombinant FXa variant with modifications that make it inactive, thus maintaining a high-affinity binding directed at FXa inhibitors [107,108]. Another study underway in humans involves the molecule PER977, which binds to oral thrombin and FXa inhibitors in a non-covalent way; this helps by preventing the anticoagulant effect of the drugs [107].

Renal and Hepatic Impairment

DOACs should be adjusted in case of renal impairment or to be safe to avoid using them. The kidneys excrete most DOACs, and any impaired kidney function can alter their levels in the blood. In the case of dabigatran, special attention should be given while prescribing since 80% of it is excreted via kidneys [106].

Patients with mild renal impairment can be prescribed rivaroxaban without any dose adjustment. In contrast, in those with moderate renal impairment, the dose should be reduced from 20 mg to 15 mg OD since the risk of bleeding outweighs the risk of recurrent thrombosis; and in case of severe renal impairment, a study was conducted to analyze the safety profile and pharmacological properties of apixaban, and no dose adjustment was required based on renal function [109].

Hepatic impairment affects the use of DOACs for two reasons: hepatic metabolism of the direct FXa inhibitors gets affected, and, second, because moderate to severely impaired liver function can affect coagulation. Rivaroxaban and apixaban are contraindicated for use in patients with clinically relevant bleeding risk, including cirrhotic patients classified as Child-Pugh B and C and anyone with severe coagulopathy. Apixaban could be considered for use in mild or moderate impaired liver function, but with caution. Dabigatran is contraindicated in patients with elevated liver enzymes and those with severe liver disease [110].

Pregnancy and Breastfeeding

DOACs should be avoided in pregnancy and breastfeeding, and therefore, special care should be given when prescribing anticoagulation to women of the childbearing age group [103]. Bleeding complications along with reproductive toxicity have been noticed in animal models receiving rivaroxaban, dabigatran, and apixaban [111]. Since there are no data available on the use of DOACs in pregnancy, these should be avoided in this setting.

## Conclusions

Due to the “normalization” of DOAC usage for prevalent coagulation diseases, DOAC use in uncommon clotting disorders like APS is being considered widely of late. Anticoagulation is utilized as a secondary thromboembolism prevention strategy in APS. There has been an increase in the use of DOACs in APLS in recent years, despite the lack of large-scale research proving their safety or effectiveness in this disease. This is due in large part to the rarity of APS patients compared to other anticoagulation-related diseases, such as non-valvular AF. However, despite its risks, the benefits of DOACs include decreased frequency of bleeding events, quick action, lack of dietary restrictions, and lesser interactions with medications and alcohol. DOACs also require lesser monitoring as compared to VKAs like warfarin, therefore enhancing the patient's quality of life. Rivaroxaban has the potential to be an effective and convenient alternative to warfarin in thrombotic APS patients with a single venous thromboembolism event requiring standard-intensity anticoagulation, according to the RAPS RCT, which used a laboratory surrogate primary outcome measure. We recommend further trials including RCTs in the usage of DOACs as primary thromboprophylactic agents in APS, as this could be a potential replacement for warfarin and would reduce the burden on the primary care providers for frequent INR monitoring.

## References

[1] Bick RL, Arun B, Frenkel EP (1999). Antiphospholipid-thrombosis syndromes. Haemostasis.

[2] Cohen H, Efthymiou M, Isenberg DA (2018). Use of direct oral anticoagulants in antiphospholipid syndrome. J Thromb Haemost.

[3] Duarte-García A, Pham MM, Crowson CS (2019). The epidemiology of antiphospholipid syndrome: a population-based study. Arthritis Rheumatol.

[4] Rodríguez-Pintó I, Espinosa G, Erkan D, Shoenfeld Y, Cervera R (2018). The effect of triple therapy on the mortality of catastrophic anti-phospholipid syndrome patients. Rheumatology (Oxford).

[5] Cervera R, Piette JC, Font J (2002). Antiphospholipid syndrome: clinical and immunologic manifestations and patterns of disease expression in a cohort of 1,000 patients. Arthritis Rheum.

[6] Farmer-Boatwright MK, Roubey RA (2009). Venous thrombosis in the antiphospholipid syndrome. Arterioscler Thromb Vasc Biol.

[7] Miyakis S, Lockshin MD, Atsumi T (2006). International consensus statement on an update of the classification criteria for definite antiphospholipid syndrome (APS). J Thromb Haemost.

[8] Meroni PL, Borghi MO, Raschi E, Tedesco F (2011). Pathogenesis of antiphospholipid syndrome: understanding the antibodies. Nat Rev Rheumatol.

[9] Cohen H, Cuadrado MJ, Erkan D (2020). 16th International congress on antiphospholipid antibodies task force report on antiphospholipid syndrome treatment trends. Lupus.

[10] Taraborelli M, Reggia R, Dall'Ara F (2017). Longterm outcome of patients with primary antiphospholipid syndrome: a retrospective multicenter study. J Rheumatol.

[11] Eriksson BI, Quinlan DJ, Weitz JI (2009). Comparative pharmacodynamics and pharmacokinetics of oral direct thrombin and factor xa inhibitors in development. Clin Pharmacokinet.

[12] Fields RA, Toubbeh H, Searles RP, Bankhurst AD (1989). The prevalence of anticardiolipin antibodies in a healthy elderly population and its association with antinuclear antibodies. J Rheumatol.

[13] Andreoli L, Chighizola CB, Banzato A, Pons-Estel GJ, Ramire de Jesus G, Erkan D (2013). Estimated frequency of antiphospholipid antibodies in patients with pregnancy morbidity, stroke, myocardial infarction, and deep vein thrombosis: a critical review of the literature. Arthritis Care Res (Hoboken).

[14] Petri M (2000). Epidemiology of the antiphospholipid antibody syndrome. J Autoimmun.

[15] Mehrani T, Petri M Epidemiology of the antiphospholipid syndrome. Handbook of Systemic Autoimmune Diseases.

[16] Biggioggero M, Meroni PL (2010). The geoepidemiology of the antiphospholipid antibody syndrome. Autoimmun Rev.

[17] Cervera R (2017). Antiphospholipid syndrome. Thromb Res.

[18] Rodríguez-Pintó I, Moitinho M, Santacreu I, Shoenfeld Y, Erkan D, Espinosa G, Cervera R (2016). Catastrophic antiphospholipid syndrome (CAPS): descriptive analysis of 500 patients from the International CAPS Registry. Autoimmun Rev.

[19] Cervera R, Espinosa G, Bucciarelli S, Gómez-Puerta JA, Font J (2006). Lessons from the catastrophic antiphospholipid syndrome (CAPS) registry. Autoimmun Rev.

[20] Finazzi G (2001). The epidemiology of the antiphospholipid syndrome: who is at risk?. Curr Rheumatol Rep.

[21] Girón-González JA, García del Río E, Rodríguez C, Rodríguez-Martorell J, Serrano A (2017). Antiphospholipid syndrome and asymptomatic carriers of antiphospholipid antibody: prospective analysis of 404 individuals. J Rheumatol.

[22] Yasuda M, Takakuwa K, Tokunaga A, Tanaka K (1995). Prospective studies of the association between anticardiolipin antibody and outcome of pregnancy. Obstet Gynecol.

[23] Allen JY, Tapia-Santiago C, Kutteh WH (1996). Antiphospholipid antibodies in patients with preeclampsia. Am J Reprod Immunol.

[24] van Pampus MG, Dekker GA, Wolf H, Huijgens PC, Koopman MM, von Blomberg BM, Büller HR (1999). High prevalence of hemostatic abnormalities in women with a history of severe preeclampsia. Am J Obstet Gynecol.

[25] Roldan V, Lecumberri R, Muñoz-Torrero JF, Vicente V, Rocha E, Brenner B, Monreal M (2009). Thrombophilia testing in patients with venous thromboembolism. Findings from the RIETE registry. Thromb Res.

[26] Lockshin MD, Druzin ML, Goei S, Qamar T, Magid MS, Jovanovic L, Ferenc M (1985). Antibody to cardiolipin as a predictor of fetal distress or death in pregnant patients with systemic lupus erythematosus. N Engl J Med.

[27] Yetman DL, Kutteh WH (1996). Antiphospholipid antibody panels and recurrent pregnancy loss: prevalence of anticardiolipin antibodies compared with other antiphospholipid antibodies. Fertil Steril.

[28] Branch DW, Silver RM, Blackwell JL, Reading JC, Scott JR (1992). Outcome of treated pregnancies in women with antiphospholipid syndrome: an update of the Utah experience. Obstet Gynecol.

[29] Hughes GR (1993). The antiphospholipid syndrome: ten years on. Lancet.

[30] Galli M, Barbui T, Zwaal RF, Comfurius P, Bevers EM (1993). Antiphospholipid antibodies: involvement of protein cofactors. Antiphospholipid antibodies: involvement of protein cofactors.

[31] Hunt J, Krilis S (1994). The ﬁfth domain of beta 2-glycoprotein I contains a phospholipid binding site (Cys281-Cys288) and a region recognized by anticardiolipin antibodies. J Immunol.

[32] Pierangeli SS, Chen PP, Raschi E (2008). Antiphospholipid antibodies and the antiphospholipid syndrome: pathogenic mechanisms. Semin Thromb Hemost.

[33] Vega-Ostertag M, Casper K, Swerlick R, Ferrara D, Harris EN, Pierangeli SS (2005). Involvement of p38 MAPK in the up-regulation of tissue factor on endothelial cells by antiphospholipid antibodies. Arthritis Rheum.

[34] Rand JH, Wu XX, Quinn AS (2010). Hydroxychloroquine protects the annexin A5 anticoagulant shield from disruption by antiphospholipid antibodies: evidence for a novel effect for an old antimalarial drug. Blood.

[35] Nagahama M, Nomura S, Kanazawa S, Ozaki Y, Kagawa H, Fukuhara S (2003). Significance of anti-oxidized LDL antibody and monocyte-derived microparticles in anti-phospholipid antibody syndrome. Autoimmunity.

[36] Di Simone N, Raschi E, Testoni C (2005). Pathogenic role of anti-beta 2-glycoprotein I antibodies in antiphospholipid associated fetal loss: characterisation of beta 2-glycoprotein I binding to trophoblast cells and functional effects of anti-beta 2-glycoprotein I antibodies in vitro. Ann Rheum Dis.

[37] Mulla MJ, Brosens JJ, Chamley LW (2009). Antiphospholipid antibodies induce a pro-inflammatory response in first trimester trophoblast via the TLR4/MyD88 pathway. Am J Reprod Immunol.

[38] Heidbuchel H, Verhamme P, Alings M (2013). EHRA practical guide on the use of new oral anticoagulants in patients with non-valvular atrial fibrillation: executive summary. Eur Heart J.

[39] Mann KG, Brummel K, Butenas S (2003). What is all that thrombin for?. J Thromb Haemost.

[40] Kubitza D, Becka M, Roth A, Mueck W (2008). Dose-escalation study of the pharmacokinetics and pharmacodynamics of rivaroxaban in healthy elderly subjects. Curr Med Res Opin.

[41] Weitz JI, Eikelboom JW, Samama MM (2012). New antithrombotic drugs: antithrombotic therapy and prevention of thrombosis, 9th ed: American College of Chest Physicians Evidence-Based Clinical Practice Guidelines. Chest.

[42] Eisenberg PR, Siegel JE, Abendschein DR, Miletich JP (1993). Importance of factor Xa in determining the procoagulant activity of whole-blood clots. J Clin Invest.

[43] Perzborn E, Heitmeier S, Laux V (2015). Effects of rivaroxaban on platelet activation and platelet-coagulation pathway interaction: in vitro and in vivo studies. J Cardiovasc Pharmacol Ther.

[44] Di Nisio M, Middeldorp S, Büller HR (2005). Direct thrombin inhibitors. N Engl J Med.

[45] Hirsh J, Weitz JI (1999). New antithrombotic agents. Lancet.

[46] Bauersachs RM (2012). Use of anticoagulants in elderly patients. Thromb Res.

[47] Harter K, Levine M, Henderson SO (2015). Anticoagulation drug therapy: a review. West J Emerg Med.

[48] Holbrook A, Schulman S, Witt DM (2012). Evidence-based management of anticoagulant therapy: antithrombotic therapy and prevention of thrombosis, 9th ed: American College of Chest Physicians Evidence-Based Clinical Practice Guidelines. Chest.

[49] Crowther MA, Ginsberg JS, Julian J (2003). A comparison of two intensities of warfarin for the prevention of recurrent thrombosis in patients with the antiphospholipid antibody syndrome. N Engl J Med.

[50] Keeling D, Baglin T, Tait C (2011). Guidelines on oral anticoagulation with warfarin - fourth edition. Br J Haematol.

[51] Cohen H, Doré CJ, Clawson S, Hunt BJ, Isenberg D, Khamashta M, Muirhead N (2015). Rivaroxaban in antiphospholipid syndrome (RAPS) protocol: a prospective, randomized controlled phase II/III clinical trial of rivaroxaban versus warfarin in patients with thrombotic antiphospholipid syndrome, with or without SLE. Lupus.

[52] Moore GW (2014). Recent guidelines and recommendations for laboratory detection of lupus anticoagulants. Semin Thromb Hemost.

[53] Pengo V, Tripodi A, Reber G, Rand JH, Ortel TL, Galli M, De Groot PG (2009). Update of the guidelines for lupus anticoagulant detection. Subcommittee on Lupus Anticoagulant/Antiphospholipid Antibody of the Scientific and Standardisation Committee of the International Society on Thrombosis and Haemostasis. J Thromb Haemost.

[54] Mekaj YH, Mekaj AY, Duci SB, Miftari EI (2015). New oral anticoagulants: their advantages and disadvantages compared with vitamin K antagonists in the prevention and treatment of patients with thromboembolic events. Ther Clin Risk Manag.

[55] Katerenchuk V, Duarte GS, Martins E Pereira G (2021). Satisfaction of patients with nonvitamin K anticoagulants compared to vitamin K antagonists: a systematic review and meta-analysis. Thromb Haemost.

[56] Arachchillage DR, Efthymiou M, Mackie IJ, Lawrie AS, Machin SJ, Cohen H (2015). Rivaroxaban and warfarin achieve effective anticoagulation, as assessed by inhibition of TG and in-vivo markers of coagulation activation, in patients with venous thromboembolism. Thromb Res.

[57] Efthymiou M, Lawrie AS, Mackie I, Arachchillage D, Lane PJ, Machin S, Cohen H (2015). Thrombin generation and factor X assays for the assessment of warfarin anticoagulation in thrombotic antiphospholipid syndrome. Thromb Res.

[58] Patel MR, Mahaffey KW, Garg J (2011). Rivaroxaban versus warfarin in nonvalvular atrial fibrillation. N Engl J Med.

[59] Cervera R, Serrano R, Pons-Estel GJ (2015). Morbidity and mortality in the antiphospholipid syndrome during a 10-year period: a multicentre prospective study of 1000 patients. Ann Rheum Dis.

[60] Pengo V, Denas G, Zoppellaro G (2018). Rivaroxaban vs warfarin in high-risk patients with antiphospholipid syndrome. Blood.

[61] Cohen H, Hunt BJ, Efthymiou M (2016). Rivaroxaban versus warfarin to treat patients with thrombotic antiphospholipid syndrome, with or without systemic lupus erythematosus (RAPS): a randomised, controlled, open-label, phase 2/3, non-inferiority trial. Lancet Haematol.

[62] Ordi-Ros J, Sáez-Comet L, Pérez-Conesa M (2019). Rivaroxaban versus vitamin K antagonist in antiphospholipid syndrome: a randomized noninferiority trial. Ann Intern Med.

[63] Dufrost V, Risse J, Reshetnyak T (2018). Increased risk of thrombosis in antiphospholipid syndrome patients treated with direct oral anticoagulants. Results from an international patient-level data meta-analysis. Autoimmun Rev.

[64] Tektonidou MG, Andreoli L, Limper M (2019). EULAR recommendations for the management of antiphospholipid syndrome in adults. Ann Rheum Dis.

[65] Arachchillage DR, Gomez K, Alikhan R, Anderson JA, Lester W, Laffan M (2020). Addendum to British Society for Haematology Guidelines on investigation and management of antiphospholipid syndrome, 2012 (Br J Haematol 2012; 157: 47-58): use of direct acting oral anticoagulants. Br J Haematol.

[66] Ortel TL, Neumann I, Ageno W (2020). American Society of Hematology 2020 guidelines for management of venous thromboembolism: treatment of deep vein thrombosis and pulmonary embolism. Blood Adv.

[67] Mueck W, Lensing AW, Agnelli G, Decousus H, Prandoni P, Misselwitz F (2011). Rivaroxaban: population pharmacokinetic analyses in patients treated for acute deep-vein thrombosis and exposure simulations in patients with atrial fibrillation treated for stroke prevention. Clin Pharmacokinet.

[68] Vrijens B, Heidbuchel H (2015). Non-vitamin K antagonist oral anticoagulants: considerations on once- vs. twice-daily regimens and their potential impact on medication adherence. Europace.

[69] Testa S, Paoletti O, Legnani C (2018). Low drug levels and thrombotic complications in high-risk atrial fibrillation patients treated with direct oral anticoagulants. J Thromb Haemost.

[70] Goldhaber SZ, Eriksson H, Kakkar A (2016). Efficacy of dabigatran versus warfarin in patients with acute venous thromboembolism in the presence of thrombophilia: Findings from RE-COVER®, RE-COVER™ II, and RE-MEDY™. Vasc Med.

[71] Woller SC, Stevens SM, Kaplan DA, T Rondina M (2018). Protocol modification of apixaban for the secondary prevention of thrombosis among patients with antiphospholipid syndrome study. Clin Appl Thromb Hemost.

[72] Chaturvedi S, McCrae KR (2017). Diagnosis and management of the antiphospholipid syndrome. Blood Rev.

[73] (2021). PRAC recommendations on signals. Updated.

[74] Ruiz-Irastorza G, Cuadrado MJ, Ruiz-Arruza I (2011). Evidence-based recommendations for the prevention and long-term management of thrombosis in antiphospholipid antibody-positive patients: report of a task force at the 13th International Congress on antiphospholipid antibodies. Lupus.

[75] Dobrowolski C, Erkan D (2019). Treatment of antiphospholipid syndrome beyond anticoagulation. Clin Immunol.

[76] Erkan D, Salmon J, Lockshin M (2017). Anti-phospholipid Syndrome. Kelley and Firestein's Textbook of Rheumatology.

[77] Dentali F, Manfredi E, Crowther M, Ageno W (2005). Long-duration therapy with low molecular weight heparin in patients with antiphospholipid antibody syndrome resistant to warfarin therapy. J Thromb Haemost.

[78] Bates SM, Greer IA, Middeldorp S, Veenstra DL, Prabulos AM, Vandvik PO (2012). VTE, thrombophilia, antithrombotic therapy, and pregnancy: Antithrombotic Therapy and Prevention of Thrombosis, 9th ed: American College of Chest Physicians Evidence-Based Clinical Practice Guidelines. Chest.

[79] Rai R, Cohen H, Dave M, Regan L (1997). Randomised controlled trial of aspirin and aspirin plus heparin in pregnant women with recurrent miscarriage associated with phospholipid antibodies (or antiphospholipid antibodies). BMJ.

[80] Kutteh WH (1996). Antiphospholipid antibody-associated recurrent pregnancy loss: treatment with heparin and low-dose aspirin is superior to low-dose aspirin alone. Am J Obstet Gynecol.

[81] Empson M, Lassere M, Craig J, Scott J (2005). Prevention of recurrent miscarriage for women with antiphospholipid antibody or lupus anticoagulant. Cochrane Database Syst Rev.

[82] Sammaritano LR (2020). Antiphospholipid syndrome. Best Pract Res Clin Rheumatol.

[83] Erkan D, Harrison MJ, Levy R (2007). Aspirin for primary thrombosis prevention in the antiphospholipid syndrome: a randomized, double-blind, placebo-controlled trial in asymptomatic antiphospholipid antibody-positive individuals. Arthritis Rheum.

[84] Carmi O, Berla M, Shoenfeld Y, Levy Y (2017). Diagnosis and management of catastrophic antiphospholipid syndrome. Expert Rev Hematol.

[85] Petri M (2011). Use of hydroxychloroquine to prevent thrombosis in systemic lupus erythematosus and in antiphospholipid antibody-positive patients. Curr Rheumatol Rep.

[86] Espinola RG, Pierangeli SS, Gharavi AE, Harris EN (2002). Hydroxychloroquine reverses platelet activation induced by human IgG antiphospholipid antibodies. Thromb Haemost.

[87] Meroni PL, Raschi E, Testoni C (2001). Statins prevent endothelial cell activation induced by antiphospholipid (anti-beta2-glycoprotein I) antibodies: effect on the proadhesive and proinflammatory phenotype. Arthritis Rheum.

[88] Ferrara DE, Swerlick R, Casper K, Meroni PL, Vega-Ostertag ME, Harris EN, Pierangeli SS (2004). Fluvastatin inhibits up-regulation of tissue factor expression by antiphospholipid antibodies on endothelial cells. J Thromb Haemost.

[89] Erkan D, Vega J, Ramón G, Kozora E, Lockshin MD (2013). A pilot open-label phase II trial of rituximab for non-criteria manifestations of antiphospholipid syndrome. Arthritis Rheum.

[90] Kahn P, Ramanujam M, Bethunaickan R (2008). Prevention of murine antiphospholipid syndrome by BAFF blockade. Arthritis Rheum.

[91] Huq FY, Tvarkova K, Arafa A, Kadir RA (2011). Menstrual problems and contraception in women of reproductive age receiving oral anticoagulation. Contraception.

[92] Martinelli I, Lensing AW, Middeldorp S (2016). Recurrent venous thromboembolism and abnormal uterine bleeding with anticoagulant and hormone therapy use. Blood.

[93] Skaistis J, Tagami T (2015). Risk of fatal bleeding in episodes of major bleeding with new oral anticoagulants and vitamin k antagonists: a systematic review and meta-analysis. PLoS One.

[94] Nagarakanti R, Ezekowitz MD, Oldgren J (2011). Dabigatran versus warfarin in patients with atrial fibrillation: an analysis of patients undergoing cardioversion. Circulation.

[95] Romualdi E, Donadini MP, Ageno W (2011). Oral rivaroxaban after symptomatic venous thromboembolism: the continued treatment study (EINSTEIN-extension study). Expert Rev Cardiovasc Ther.

[96] Sciascia S, Breen K, Hunt BJ (2015). Rivaroxaban use in patients with antiphospholipid syndrome and previous venous thromboembolism. Blood Coagul Fibrinolysis.

[97] Majeed A, Goldhaber SZ, Kakkar A (2016). Bleeding events with dabigatran or warfarin in patients with venous thromboembolism. Thromb Haemost.

[98] Franchini M, Bonfanti C, Mannucci PM (2015). Management of bleeding associated with new oral anticoagulants. Semin Thromb Hemost.

[99] Breen KA, Hunt BJ (2011). The new oral anticoagulants. Clin Med (Lond).

[100] Sciascia S, Hunt BJ (2014). New oral anticoagulants in the management of venous thromboembolism: a major advance?. Eur J Vasc Endovasc Surg.

[101] (2021). Pradaxa 150 mg hard capsules. Summary of product characteristics (SPC). GmBH.

[102] Stangier J, Stähle H, Rathgen K, Fuhr R (2008). Pharmacokinetics and pharmacodynamics of the direct oral thrombin inhibitor dabigatran in healthy elderly subjects. Clin Pharmacokinet.

[103] Levi M, Moore KT, Castillejos CF (2014). Comparison of three-factor and four-factor prothrombin complex concentrates regarding reversal of the anticoagulant effects of rivaroxaban in healthy volunteers. J Thromb Haemost.

[104] Cheung YW, Barco S, Hutten BA, Meijers JC, Middeldorp S, Coppens M (2015). In vivo increase in thrombin generation by four-factor prothrombin complex concentrate in apixaban-treated healthy volunteers. J Thromb Haemost.

[105] Perzborn E, Heitmeier S, Laux V, Buchmüller A (2014). Reversal of rivaroxaban-induced anticoagulation with prothrombin complex concentrate, activated prothrombin complex concentrate and recombinant activated factor VII in vitro. Thromb Res.

[106] Pollack CV Jr, Reilly PA, Eikelboom J (2015). Idarucizumab for dabigatran reversal. N Engl J Med.

[107] Costin J, Ansell J, Laulicht B, Bakhru S, Steiner S (2014). Reversal agents in development for the new oral anticoagulants. Postgrad Med.

[108] Connolly SJ, Milling TJ Jr, Eikelboom JW (2016). Andexanet alfa for acute major bleeding associated with factor Xa inhibitors. N Engl J Med.

[109] Chang M, Yu Z, Shenker A (2016). Effect of renal impairment on the pharmacokinetics, pharmacodynamics, and safety of apixaban. J Clin Pharmacol.

[110] Graff J, Harder S (2013). Anticoagulant therapy with the oral direct factor Xa inhibitors rivaroxaban, apixaban and edoxaban and the thrombin inhibitor dabigatran etexilate in patients with hepatic impairment. Clin Pharmacokinet.

[111] Arachchillage DJ, Cohen H (2013). Use of new oral anticoagulants in antiphospholipid syndrome. Curr Rheumatol Rep.

